# Recent Advances in the Control of Endoparasites in Ruminants from a Sustainable Perspective

**DOI:** 10.3390/pathogens12091121

**Published:** 2023-09-01

**Authors:** Pedro Mendoza-de Gives, María Eugenia López-Arellano, Agustín Olmedo-Juárez, Rosa Isabel Higuera-Pierdrahita, Elke von Son-de Fernex

**Affiliations:** 1 Laboratory of Helminthology, National Centre for Disciplinary Research in Animal Health and Innocuity (CENID-SAI), National Institute for Research in Forestry, Agriculture and Livestock, INIFAP-AGRICULTURA, Jiutepec Municipality 62574, Morelos State, Mexico; aolmedoj@gmail.com; 2 Faculty of High Studies-Cuautitlán (FES-Cuautitlán), National Autonomous University of Mexico (UNAM), Cuautitlán Municipality 54714, State of Mexico, Mexico; rhiguera05@comunidad.unam.mx; 3 Teaching, Research and Extension in Tropical Livestock Center, Faculty of Veterinary Medicine and Zootechnics, National Autonomous University of Mexico, Martínez de la Torre Municipality 93600, State of Veracrúz, Mexico; elkevsdf@comunidad.unam.mx

Consumer awareness of animal welfare and environmental health has led to a plateau level of global consumption putting serious pressure on the livestock industry [1]. According to the reports of the European commission of Agriculture and Rural Development, organic farming increased by over 50% in the past decade, highlighting the global trend of consumer preferences towards organic and grazing systems [2]. Thus, in the pursuit of sustainability, farmers must face new challenges, such as the risk of cattle acquiring parasite infections, which heads the list of hazards associated to outdoor livestock farming systems [3]. Parasitic diseases caused by endoparasites are responsible for severe deterioration in animal welfare, health and performance, and are some of the most important parasitic infections affecting the livestock industry all over the world [4,5,6]. Helminths are classified into two major groups: (i) nematodes (roundworms) and platyhelminths (flatworms, including cestodes and trematodes), affecting different organ systems. However, the most prevalent parasites of grazing cattle are gastrointestinal parasitic nematodes (GIPN). Nematodes infecting the digestive tract of ruminants under grazing conditions are responsible of a severe state of malnutrition, weakness, anaemia, diminished immune response and even the death of young animals [7,8,9]. The impact of these health disorders depends on the pathogenicity of the parasites involved in the infection and worm burden. Gastroenteritis caused by GIPN, even with subclinical burdens, diminishes animal performance and, consequently, leads to economic losses of animal farming systems in many countries around the world [10,11,12,13,14]. The GIPN are organisms highly adapted to the natural conditions of their environment that developed an extraordinary capability to perpetuate their life cycles using a combined lifestyle of preparasitic stages (occurs outside the animals and comprises eggs and as free-living stages, including the infective larvae L_3_) and parasitic stages (happens inside the animals either as hypobiotic, histotrophic, pre-adult or adult stages) [15,16]. Since the middle of the last century, the development of chemical anthelmintic drugs by the pharmaceutical industry has been gaining important reputation worldwide, and the use of such drugs currently is the main strategy to control GIPN in animal farming systems. New synthetic anthelmintic molecules have become the most practical, simple and profitable approach to reduce parasitic populations in ruminants. For many decades, chemical anthelmintic drugs have been useful to diminish the undesirable consequences of parasites on both health and performance of many of the most economically valuable animal species used for food resources. Over the years, the use of these chemical drugs has become more and more frequent until farmers became highly dependent on the use of anthelmintics to maintain or improve farm productivity. Up to this point, everything seemed perfect, with a solution to all parasitic problems, until this method of control began to fail. Nowadays, it is widely known that once the animals are drenched, chemical residues are eliminated into the environment through feces and urine [17,18]. Chemical residues of anthelmintic drugs eliminated by treated animals are toxic and can affect beneficial organisms such as dung beetles, who play an essential role in decomposing organic matter and in carbon and nitrogen re-cycling, benefitting both the edaphic fauna and the microflora [19,20,21] and improving soil fertility [22]. Because soil is the final destination of chemical anthelmintic residues, these compounds can be considered as an environmental threat. Additionally, chemical residues of the administered anthelmintic drug can also remain in animal tissues and in the milk, meat and other sub-products of the livestock industry intended for human consumption, representing an imminent risk for public health [23,24]. A few decades after the discovery of pesticides and synthetic chemical anthelmintic drugs, reports about anthelmintic drug efficiency failure have become more and more frequent. This was a concern not only for farmers but also for pharmaceutical companies. The fact that anthelmintics lost their efficacy was attributed to a genetic alteration of the parasites to overcome the lethal effect of these drugs. Such phenomenon is better known as anthelmintic resistance [25,26]. The first record of the lack of efficiency of an anthelmintic drug (phenothiazine) against *Haemonchus contortus* was reported in farms in Kentucky, USA in the early 1960s [27]. Since then, anthelmintic resistance has been spreading to other countries. For example, in Mexico, in 1987, the first case of anthelmintic resistance in *H. contortus* against benzimidazoles in sheep was reported by Campos-Ruelas et al. [28]. At that time, pharmaceutical companies continued discovering new molecules that served as alternative drugs and resulted in a slower dissemination of anthelmintic resistance in herds and flocks. In addition to the synthesis of benzimidazoles, other molecules, such as imidazothiazoles, were discovered, and various anthelmintic drugs were commercially available to control GIPN. This includes ivermectins, which, because of their wide spectrum of action and wide range of blank parasites, including endo- and ectoparasites, facilitated a revolutionary therapy benefiting the livestock industry worldwide [29]. Beyond those findings, new molecules, such as moxidectin and monepantel, among others, were released and used in combination to enhance their deworming effect [30]. However, irrespective of the case, it seems that any new anthelmintic drugs can generate resistance in the parasites [31]. Pharmaceutical companies have noticed that any new anthelmintic molecule is under the imminent risk of developing resistance, and herds and flocks all over the world are defenseless under the imminent risk not only to anthelmintic resistance (AR) but to the worst kind of resistance threatening the global livestock industry, namely multiple AR [32,33]. This growing problem is motive for an intense analysis of scientists trying to develop a better understanding of the nematode genes responsible for the regulation of resistance traits. So far, studies have identified several groups of genes upregulated in *H. contortus* isolates, some of which are correlated to a resistance phenotype against different anthelmintic drugs such as ivermectin or benzimidazoles [34,35]. In pursuit of reverting the anthelmintic resistance in *H. contortus*, recent studies have focused on gene expression analysis to explore the potential use of transcriptomic data for the manipulation of genes associated to ivermectin resistance mechanisms [1,36,37]. However, the number of studies on novel strategies to control GIPN using an approach different from that of the use of synthesized chemical anthelmintic drugs has increased over the last decade, with promising strategies for GIPN control [16,38]. Some of these strategies include vaccination to enhance the immunological status of animals against parasite infection [39,40], focusing on the use of antigens obtained from the intestine cells of adult parasitic nematodes such as *H. contortus* to induce immune responses that provide protection [41]. Furthermore, the use of recombinant antigen technologies has also achieved a solid immune protection of flocks against this important parasite [42]. Another alternative is the use of biological control strategies employing natural antagonists of nematodes, such as nematophagous fungi, to control parasitic nematodes affecting crops of high commercial value [43] and ruminant parasitic nematodes, using the fungus *Duddingtonia flagrans* [44]. This species is able to spontaneously produce a large number of resistant spores (chlamydospores) that can be added to the diet of the animals either in multinutritional pellets [45,46] mixed with animal feed [47] or in an aqueous oral suspension [48]. Once chlamydospores pass through the gastrointestinal tract of animals, they are expelled together with the feces to the soil and pasture. In the feces, the spores germinate, form trapping devices through their mycelia and capture, kill and feed on infective larvae, diminishing their populations in the grassland and avoiding massive and continuous self-reinfections [49,50,51,52]. Another strategy recently explored is the use of natural compounds produced by edible mushrooms [53,54] and fungi with nematocidal activity [55,56,57,58], which, in the near future, are expected to replace, either fully or partially, chemically synthesized anthelmintic drugs routinely used in farms maintained under grazing systems. In recent years, other approaches, such as the use of bioactive plants for GIN control, have been widely explored [59,60,61]. Ethnoveterinary medicine is an ancient practice performed mostly by rural communities worldwide, which has emerged as a necessity to counteract anthelmintic resistance and the negative impacts of GIPN on animal welfare and performance [62,63]. Tropical and temperate legumes represent a suitable option for anthelmintic (AH)-like activity screening due to their high content and diversity of plant secondary metabolites (PSM) with potential AH-like activity. Several plant species rich in secondary metabolites and belonging to different families have been assessed against gastrointestinal parasites, with promising results. Some secondary compounds, such as condensed tannins and hydroxycinnamic acid derivatives and coumarins, are potential alternatives for controlling these parasites [64,65]. The use of plants rich in these compounds as part of the diet for livestock under a nutraceutical approach could contribute to diminishing the parasite population in grazing animals [66,67]. Several studies, both in vitro and in vivo, have confirmed the use of PSM for GIPN control [60,61,62,63,64,65,66,67,68,69]. As the versatility of bioactive plants is one of their most appealing characteristics, they can be used as (i) a phytochemical source for drug development, (ii) as source of plant extracts or essential oils for animal drenching or (iii) as an addition to feedstuff with nutraceutical properties [70]. Among other alternatives to counteract parasitism in livestock, there is immunonutrition, which refers to the use of nutritional strategies based on dietary supplementation based on protein or energy feedstuffs to improve host immune responses to parasite infections [71]. Such supplementation induces lambs naturally infected with GIN to acquire resistance and resilience [72], facilitates the selection of animals that have developed genetic resistance against GIPN [73,74], enables the identification of resilient animals through targeted selective deworming such as the FAMACHA^®^ technique and the body score [75,76]. Other strategies include the use of a grazing management system that originally was designed to maximize the use of the main forage resource, and this technology was used to reduce the exposure time of animals to large numbers of GIN-infective larvae in grazing lands [3,77]. The use of copper oxide particles which affect *H. contortus* adult female fertility is useful for GIPN control either alone [78] or in combination with anthelmintics such as closantel, which can increase the immune and antioxidant response in lambs experimentally infected with *H. contortus* [79].

In recent years, the number of studies on novel approaches for parasite control has increased, with focus on viable tools to design an efficient integrated program for GIPN control. The inevitable new era of anthelmintic resistance in livestock, along with consumer awareness of animal welfare and environmental health, calls for in-depth scientific work to offer solutions to the livestock industry. In the pursuit of a chemically synthesized anthelmintic drug independency, parasite control, in the near future, will rely on prevention and on the combined use of strategies. The present Pathogens Special Issue aims to describe for the readers some of the novel approaches for GIPN control in ruminants, developed based on animal welfare awareness to tackle this group of pathogens in an environmentally friendly manner.
